# Supplementary material for: Whole-Genome Omics Elucidates the Role of CCM1 and Progesterone in Cerebral Cavernous Malformations within CmPn Networks
Source: Diagnostics (Basel). 2024 Aug 28;14(17):1895. doi: 10.3390/diagnostics14171895 (PMC11394482; doi:10.3390/diagnostics14171895)

Whole-Genome Omics Elucidates the Role of CCM1 and Progesterone in Cerebral Cavernous Malformations within CmPn Networks

Jacob Croft[1], Brian Grajeda[2], Liyuan Gao[3], Johnathan Abou-Fadel[1], Ahmed Badr[4], Victor Sheng[3], and Jun Zhang[1,*]

Departs of Molecular & Translational Medicine (MTM)[1]
Texas Tech University Health Science Center El Paso (TTUHSCEP), El Paso, TX 79905 USA
Department of Biological Sciences[2], University of Texas at El Paso, El Paso, TX 79902 USA
Department of Computer Sciences[3], Texas Tech University, Lubbock, TX 79409 USA
Department of Anesthesiology[4], Ochsner LSU Health Shreveport, LA 71130 USA

<u>Supplemental Materials</u>

++All correspondence:
Jun Zhang, Sc.D., Ph.D.
Department of Molecular and Translational Medicine (MTM)
Texas Tech University Health Science Center El Paso
5001 El Paso Drive, El Paso, El Paso, TX 79905
Tel: (915) 215-4197, Email: jun.zhang2000@gmail.com

Supplemental Legends

Supplemental Figure 1. Differentially Expressed Proteins (DEPs) Profiles Across Three CCM1 Genotypes. Volcano plots were used to illustrate the profiles and differential expression levels of differentially expressed proteins (DEPs) for three different comparative CCM1 genotype-pairs: **A**. WT/96 (left); **B.** KO/WT (middle), and **C.** KO/96 (right). All samples were processed and analyzed in triplicate, and significance was determined using a student's t-test with a p-value cut-off of <0.05. Upregulated DEPs with both p-value and fold change significance are represented by red points, while downregulated DEPs with both p-value and fold change significance are shown as blue points. Non-differentially expressed proteins (non-DEPs) are represented by light, medium, and dark grey points.

Supplemental Figure 2. Profiles of Differentially Expressed Proteins (DEPs) and Genes (DEGs) Under Three CCM1 Levels in Response to PRG Actions. Volcano plots were used to illustrate the differential expression profiles of three different CCM1 genotypes in response to PRG actions: **A.** Differentially Expressed Proteins (DEPs) Profiles in Response to PRG Actions Across Three CCM1 Genotypes: KO (left), WT (middle), and 96 (right). **B.** Differentially Expressed Genes (DEGs) Profiles in Response to PRG Actions Across Two CCM1 Genotypes: KO (left), and 96 (right). All samples were processed and analyzed in triplicate, and significance was determined using a student's t-test with a p-value cut-off of <0.05. Upregulated DEPs with both p-value and fold change significance are represented by red points, while downregulated DEPs with both p-value and fold change significance are shown as blue points. Non-differentially expressed proteins (non-DEPs) are represented by light, medium, and dark grey points. **C-D.** overrepresentation analysis (ORA) of comparative omics into signaling pathways for MEF cells in response to PRG actions through CCM1. In this panel, pathway enrichments were performed using Gene Ontology (GO) analysis of identified groups of DEPs/DEGs to determine the statistical significance of biological processes and enrich their associated molecular pathways. These molecular pathways indicate the presence of DEPs among three CCM1 genotypes (C) and DEGs among two CCM1 genotypes (D) in response to PRG actions. **E-F.** To further

evaluate these identified signaling pathways through GO enrichment analysis, , ORA analysis along with KEGG enrichment were conducted under similar conditions for DEPs/DEGs among three CCM1 genotypes (**E**) and DEGs among two genotypes in response to PRG actions (**F**).

Supplemental Figure 3. Pathway enrichment from DEPs and DEGs from MEF cells responding to PRG actions. Identifying Pathways Associated with Progesterone actions in general, then for non-mPR-Specific Progesterone Actions the secondary filters. **A.** This diagram shows our pull-down approach to select pathways either bound to the first filter (all progesterone actions) or passing through the second filter (for selection of mPR-specific progesterone actions), focusing on progesterone (PRG) actions not mediated by mPR. Using the filter dataset from mifepristone (MIF, as an antagonist), we identified pathways shared by the glucocorticoid receptor (GR) and the nuclear progesterone receptor (nPR). Notably, MIF functions as an agonist and works synergistically with PRG on mPR-mediated signaling. **B-C:** Omics analysis of DEPs and RNA differentially expressed RNAs (DEGs) in MEF cells responding to PRG action was conducted across three CCM1 genotypes: CCM1-KO (complete depletion), CCM1-WT (natural low expression), and CCM1-96 (ectopic overexpression). The study encompassed both proteomic (**B**) and RNAseq data (**C**) to explore the effects of PRG actions on protein and RNA expression profiles in these different genetic backgrounds. **D-E.** Volcano plots were utilized to visualize the expression profiles and differential expression levels of DEPs (**D**) across three distinct CCM1 genotypes, and DEGs (**E**) across two CCM1 genotypes, in response to PRG actions. **F-G.** Heatmaps were employed to depict the expression profiles and differential expression levels of DEPs (**F**) among three different CCM1 genotypes, and DEGs (**G**) among two CCM1 genotypes, in response to PRG actions. All samples were processed and analyzed in triplicate, with statistical significance determined using a student's t-test ($p < 0.05$). Upregulated DEPs meeting both p-value and fold change criteria are denoted by red points, while downregulated DEPs meeting these criteria are indicated by blue points. Non-differentially expressed proteins (non-DEPs) are represented by varying shades of grey points.   **H-I.** ORA analysis of omics data was conducted to delineate signaling pathways in MEF cells triggered by PRG actions through CCM1. Pathway enrichments were assessed using Gene Ontology (GO) analysis of identified groups of differentially expressed proteins (DEPs) and genes (DEGs) to ascertain the statistical significance of biological processes and enhance their associated molecular pathways. These pathways highlight the presence of DEPs across three CCM1 genotypes (**H**) and DEGs across two CCM1 genotypes (**I**) in response to

PRG actions. **J-K.** Similarly, to further assess these identified signaling pathways through GO enrichment analysis, ORA analysis along with KEGG enrichment was performed under similar conditions for DEPs/DEGs among three CCM1 genotypes (**J**) and DEGs among two genotypes in response to PRG actions (K).

Supplemental Figure 4. Pathway enrichment from DEPs and DEGs of MEF cells response to mPR- specific PRG actions. Using our two filter pull-down systems, we can identify pathways associated with mPR-specific progesterone actions. **A-B.** Omics analysis of DEPs and DEGs followed by filtration in MEF cells across three CCM1 genotypes—CCM1-KO (complete depletion), CCM1-WT (natural low expression), and CCM1-96 (ectopic overexpression)—responding to mPR-specific PRG action was conducted. The study incorporated both proteomic (**A**) and RNAseq data (**B**) to examine the effects of mPR-specific PRG actions on protein and RNA expression profiles in these different genetic backgrounds. **C-D.** Heatmaps were utilized to illustrate the expression profiles and differential expression levels of DEPs (**C**) among three different CCM1 genotypes and DEGs (**D**) among two CCM1 genotypes in response to mPR-specific PRG actions. All samples were processed and analyzed in triplicate, with statistical significance determined using a Student's t-test ($p < 0.05$). Upregulated DEPs meeting both p-value and fold change criteria are marked by red points, while downregulated DEPs meeting these criteria are indicated by blue points. Non-differentially expressed proteins (non-DEPs) are represented by varying shades of grey points. **E-F.** ORA analysis of omics data was performed to delineate signaling pathways in MEF cells triggered by mPR-specific PRG actions through CCM1. Pathway enrichments were evaluated using Gene Ontology (GO) analysis of identified groups of differentially expressed proteins (DEPs) and genes (DEGs) to determine the statistical significance of biological processes and enrich their associated molecular pathways. These pathways highlight the presence of DEPs across three CCM1 genotypes (**E**) and DEGs across two CCM1 genotypes (**F**) in response to mPR-specific PRG actions. **G-H.** Similarly, to further evaluate these identified signaling pathways through GO enrichment analysis, ORA analysis along with KEGG enrichment was conducted under similar conditions for DEPs/DEGs among three CCM1 genotypes (**G**) and DEGs among two genotypes in response to mPR-specific PRG actions (**H**).

Suppl. Figure 1: Differentially Expressed Genes(DEGs) Protein profiles under 3 different levels of CCM1

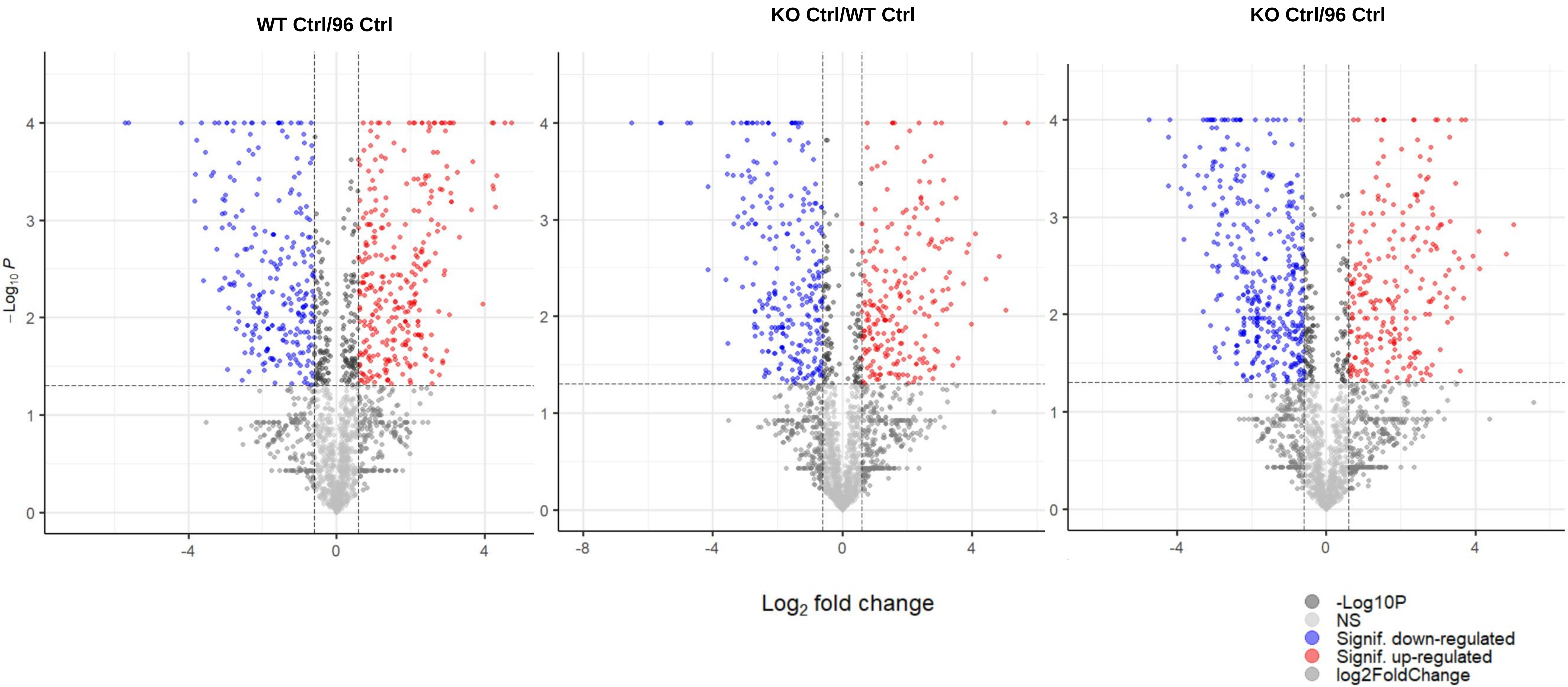

Suppl. Figure 2A:Differentially expressed proteins (DEPs) under varying levels of CCM1 Expression in response to PRG treatment

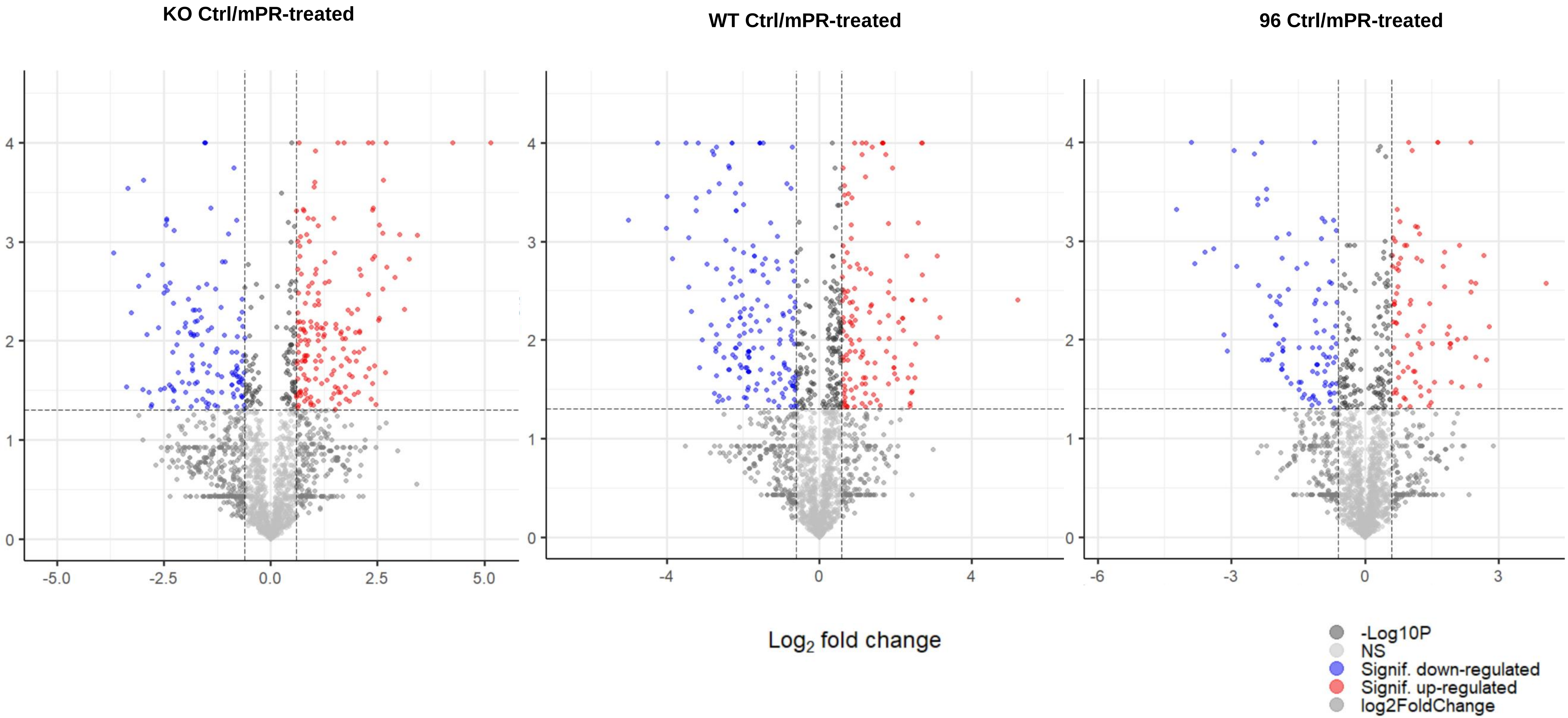

Suppl. Figure 2B: RNA Expression of MEF KO and MEF 96 in response to PRG Actions

MEF KO-Ctrl/MP__Poisson Distribution

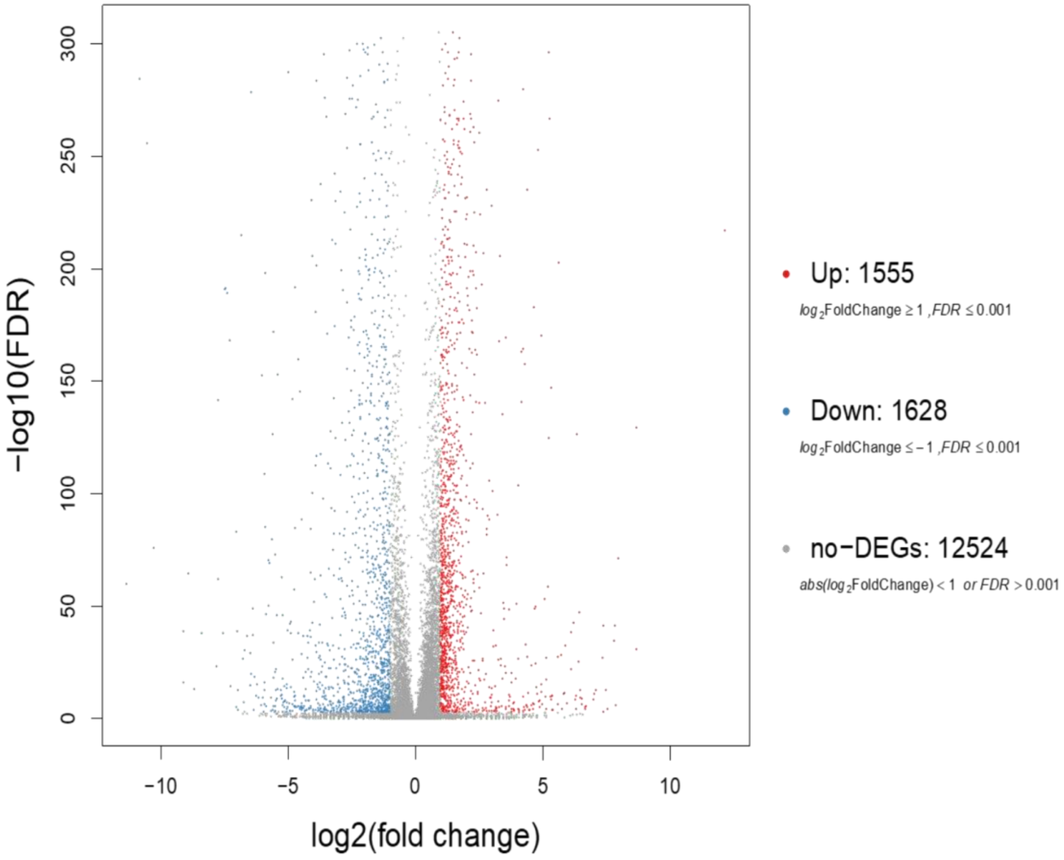

MEF 96-Ctrl/MP__Poisson Distribution

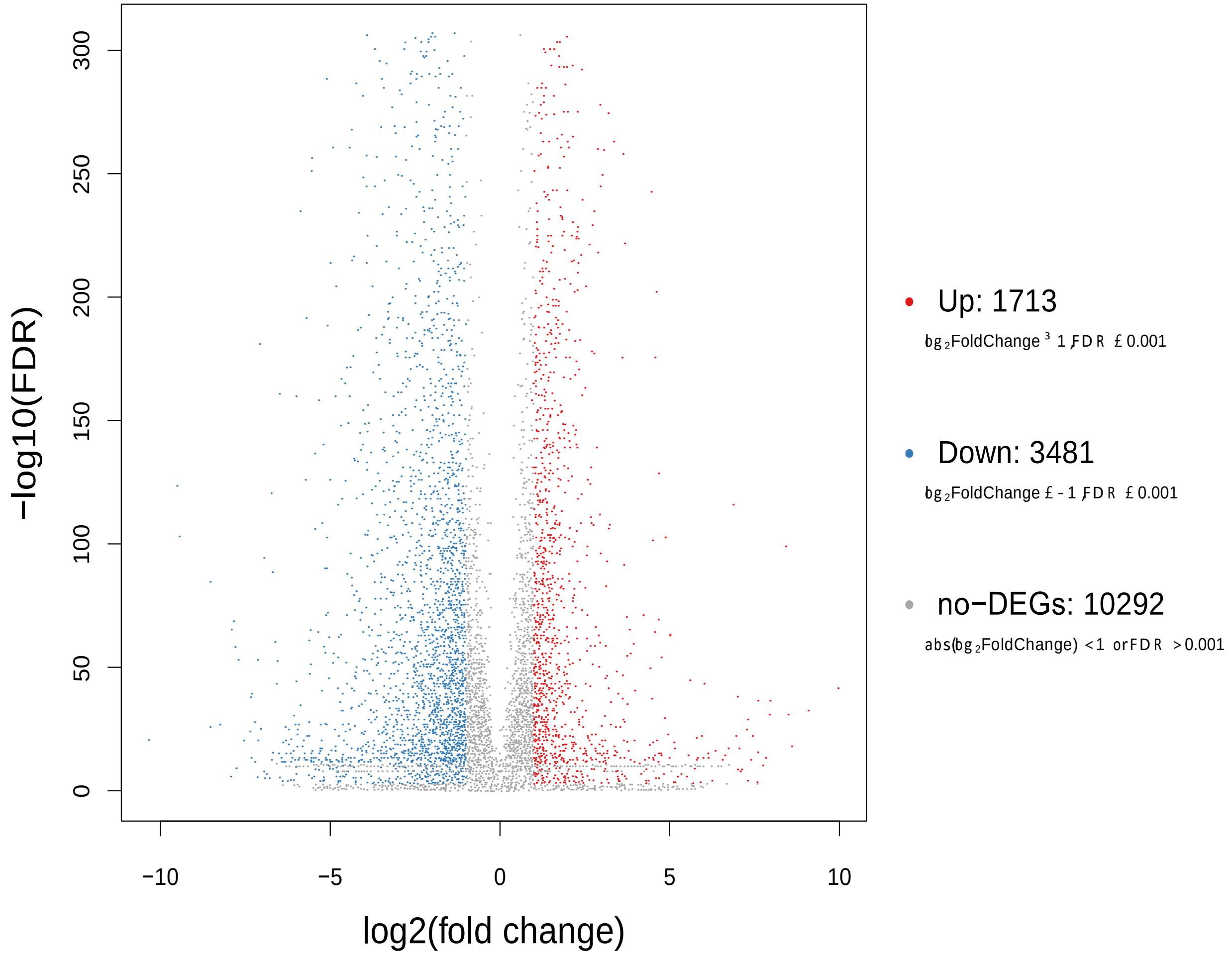

2C. Proteomic GO

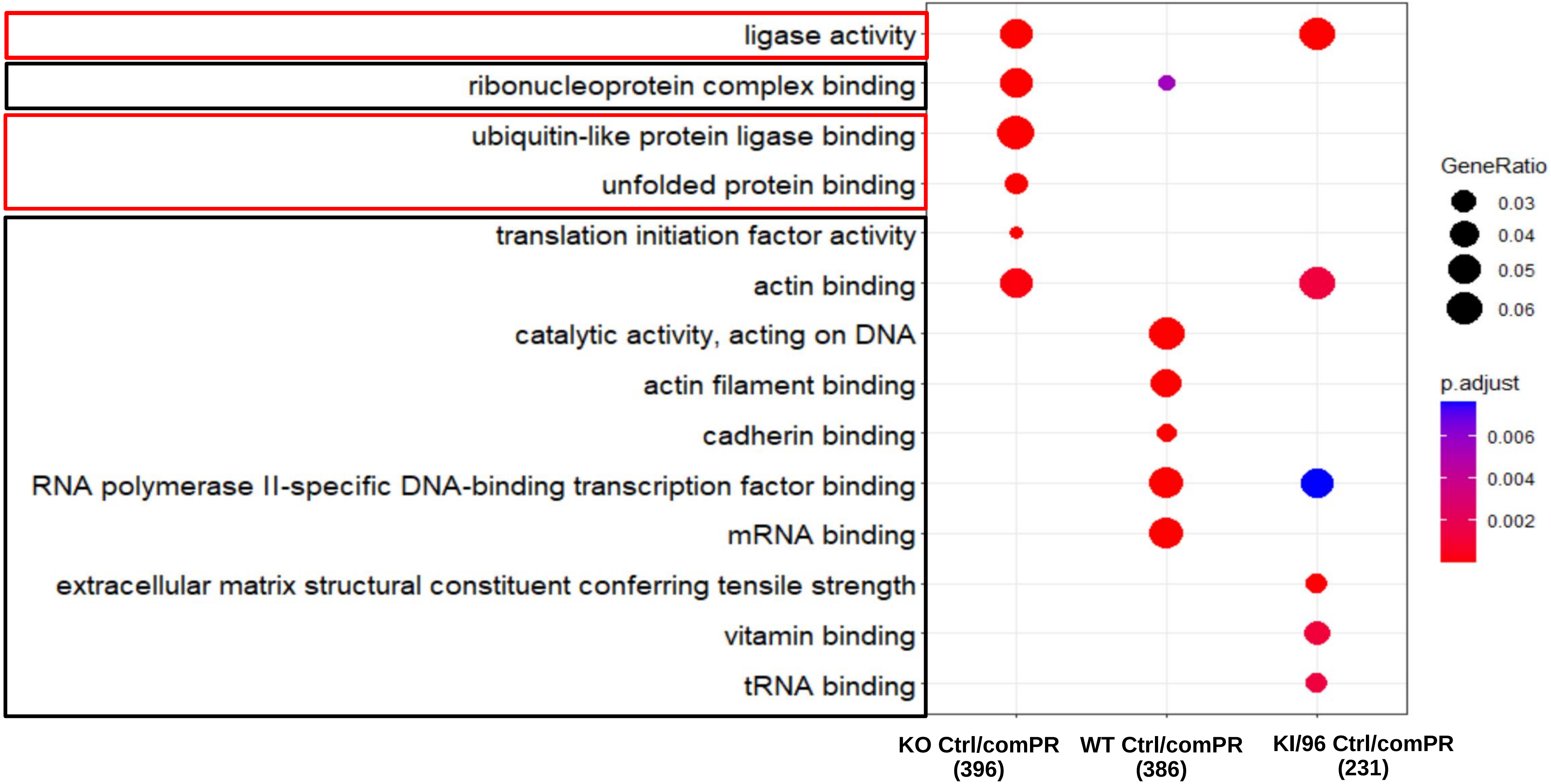

2D. RNAseq GO

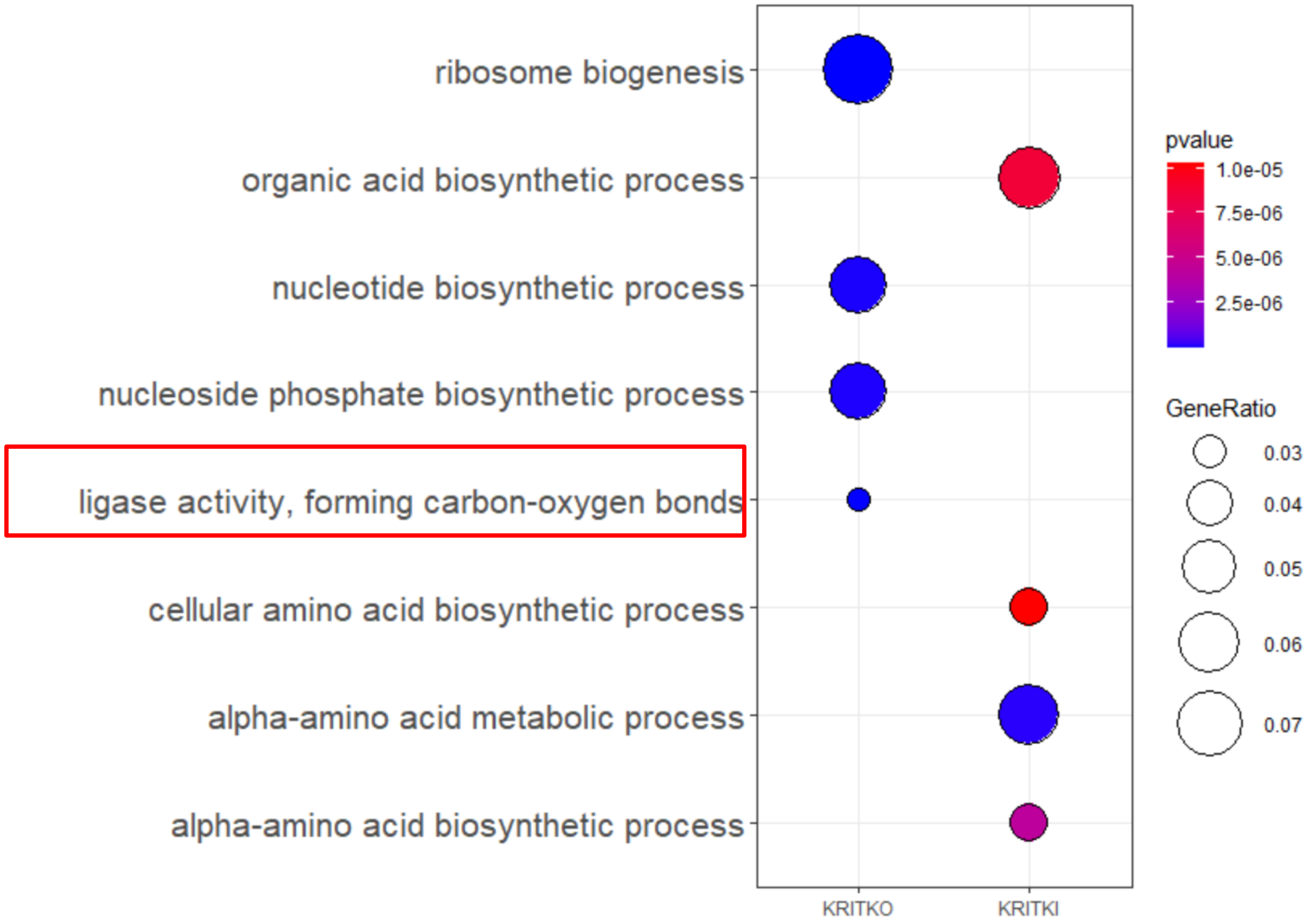

ribosome biogenesis
organic acid biosynthetic process
nucleotide biosynthetic process
nucleoside phosphate biosynthetic process
ligase activity, forming carbon-oxygen bonds
cellular amino acid biosynthetic process
alpha-amino acid metabolic process
alpha-amino acid biosynthetic process
KRITKO
KRITKI
pvalue
1.0e-05
7.5e-06
5.0e-06
2.5e-06
GeneRatio
0.03
0.04
0.05
0.06
0.07

2E. Proteomic KEGG

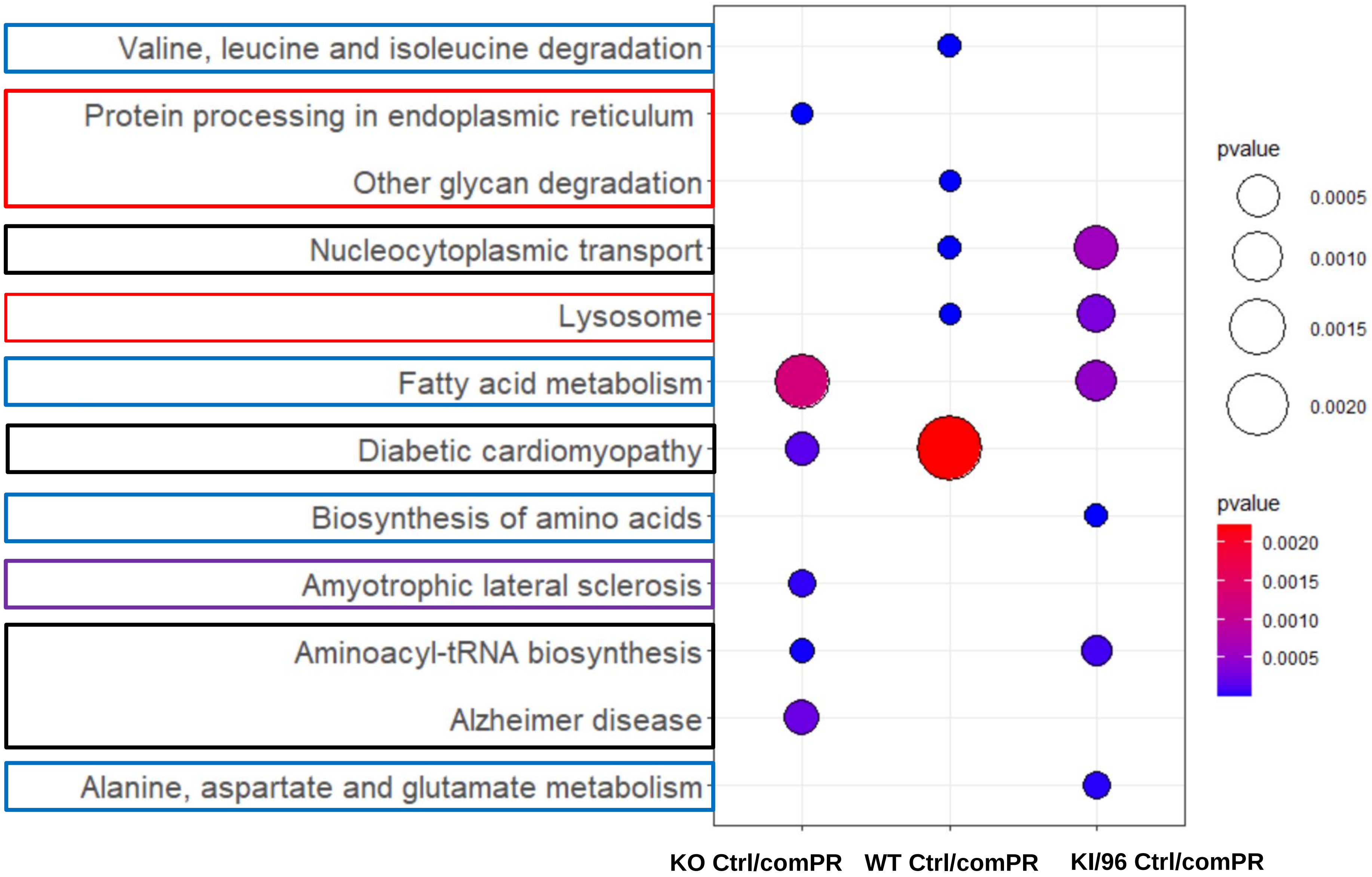

KEGG

2F. RNAseq KEGG

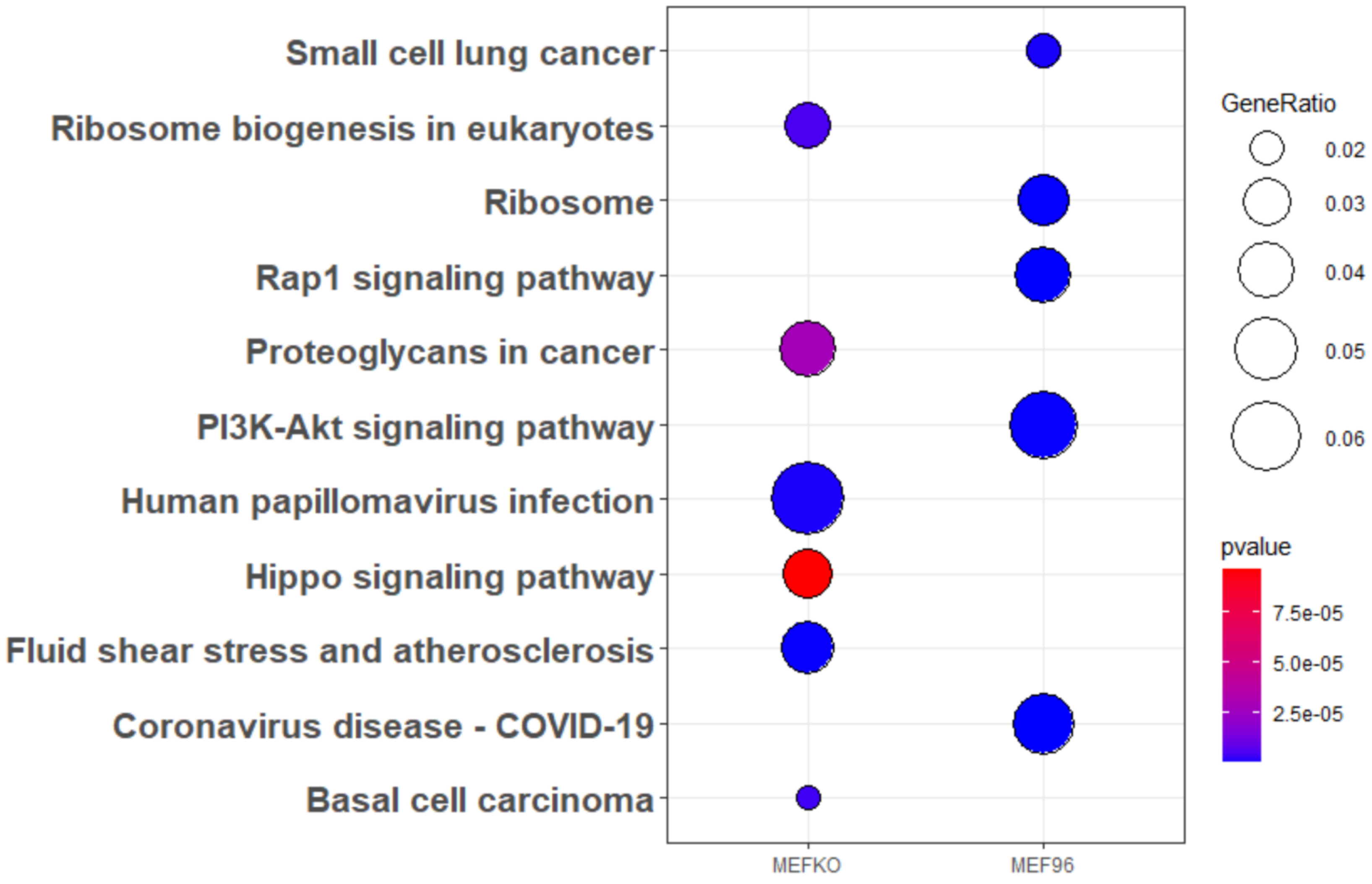

Suppl. Figure 3A:
Analysis Scheme

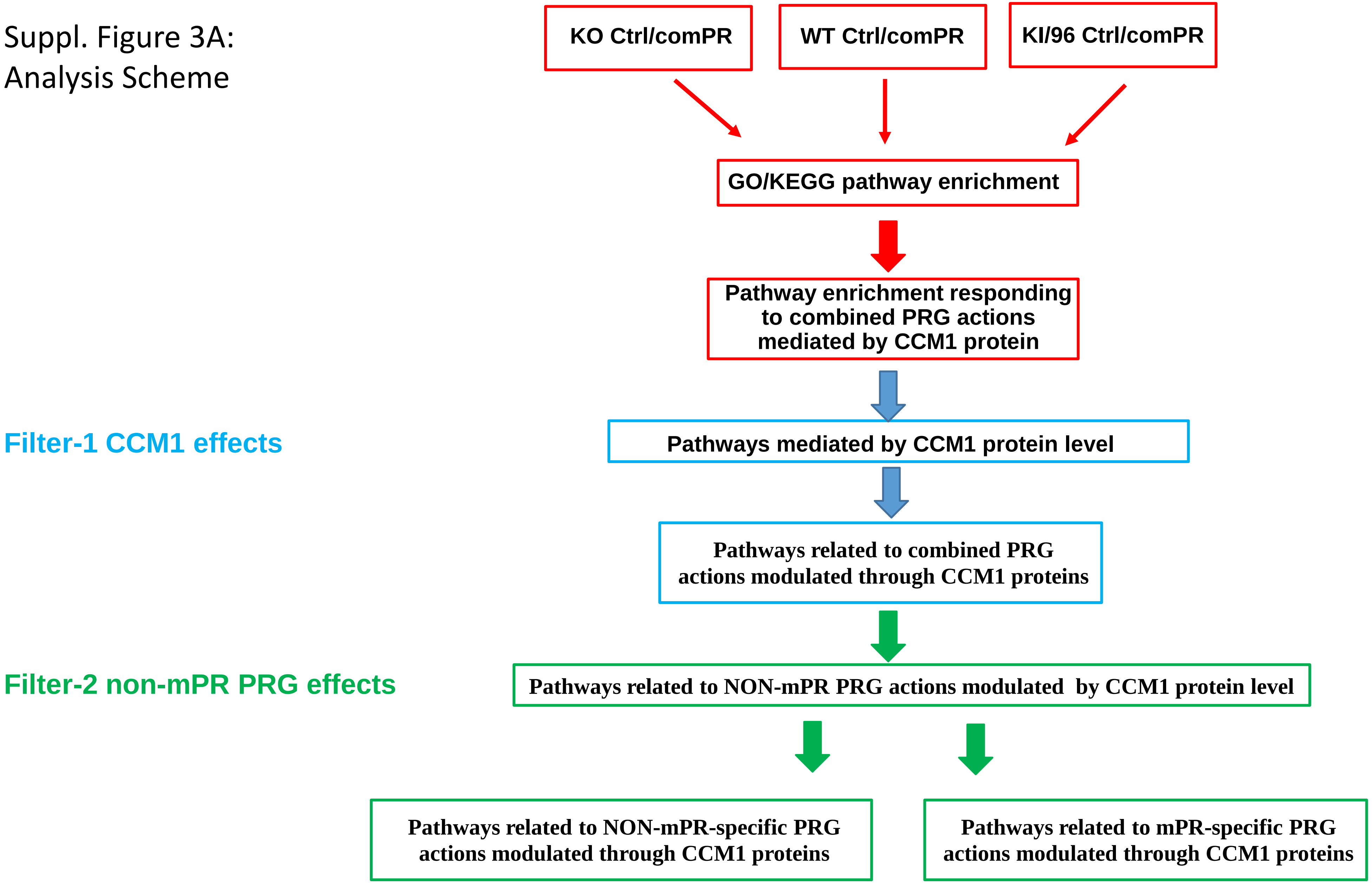

Suppl. Figure 3B-C: Omnics analysis of DEP and differentially expressed RNA for non-mPR response to PRG

3B.Protein non-mPR response to PRG under different levels of CCM1

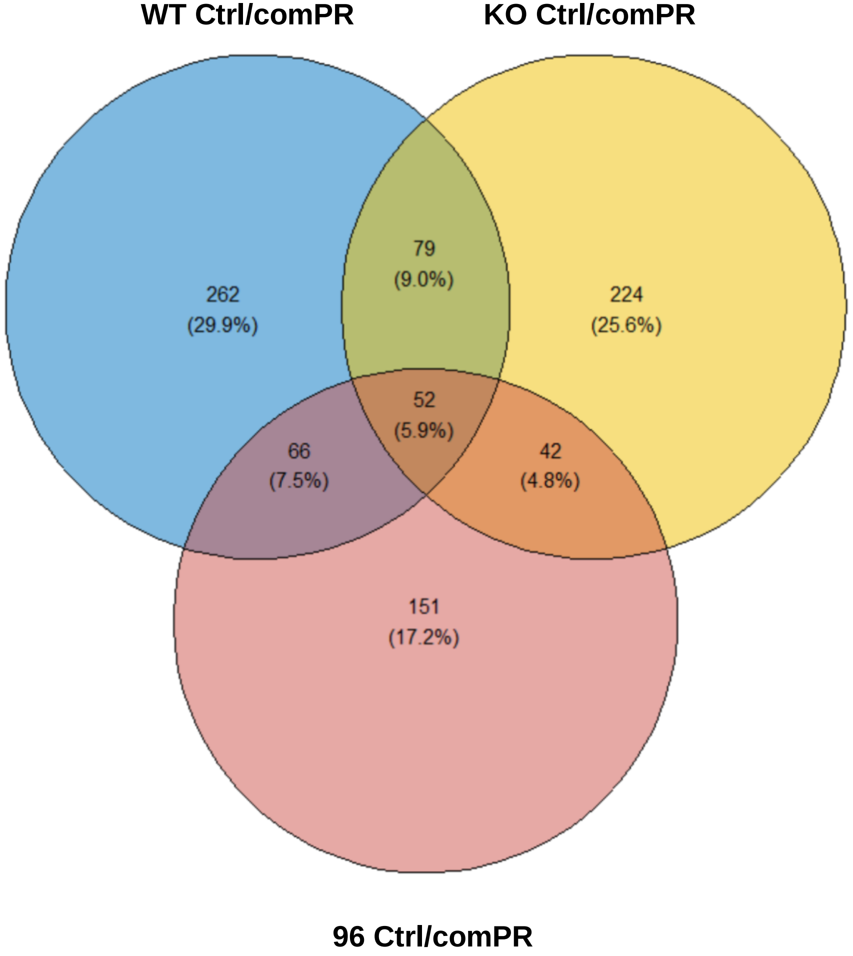

3C. Comparative RNA data expressions between CCM1-KO and CCM1 KI_96 models in response to non-mPR progesterone actions

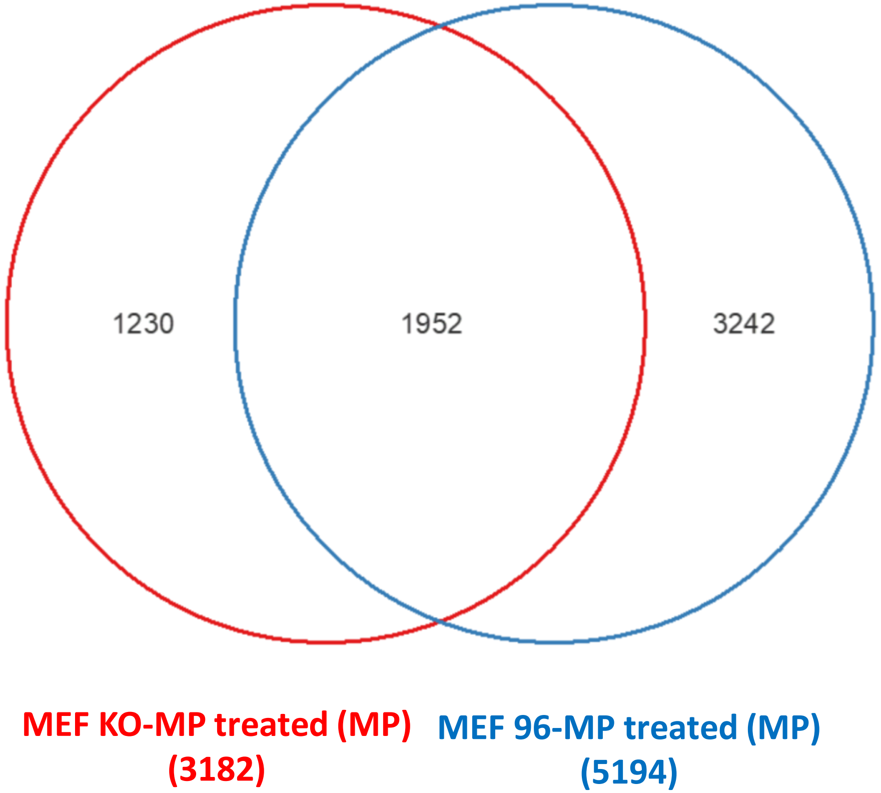

Suppl. Figure 3D: Differentially expressed proteins (DEPs) non-mPR specific response to progesterone

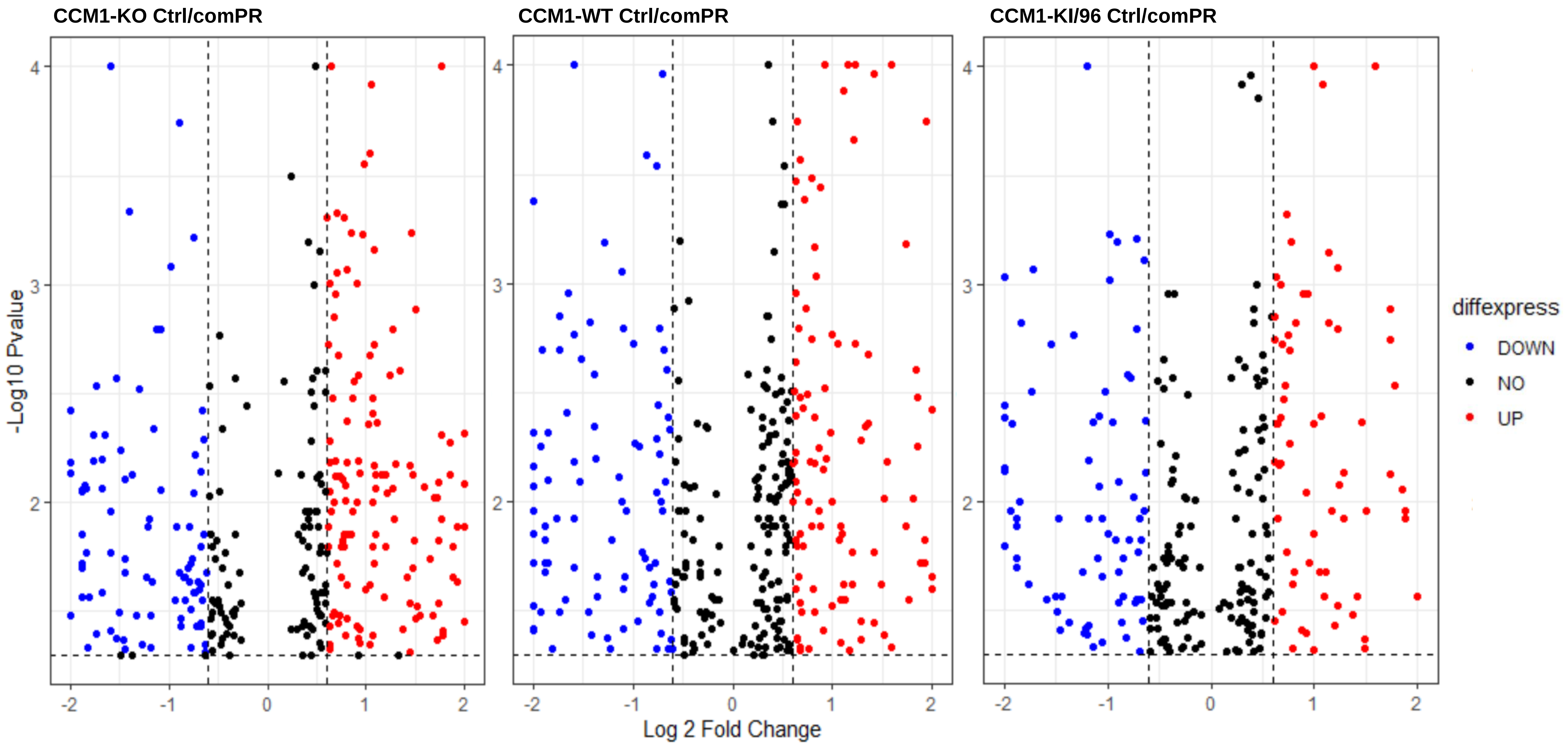

Suppl. Figure 3E: Differentially expressed RNAseq under two levels of CCM1 expression for non-mPR responses to PRG

KO Ctrl/KO Treated

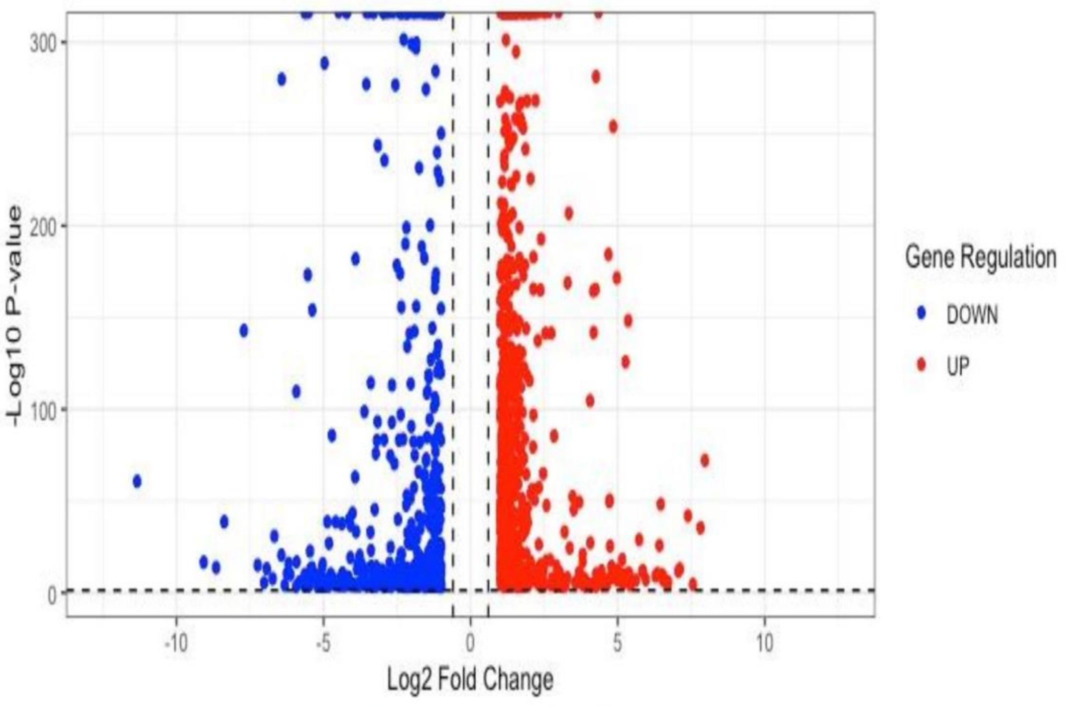

MEF96 Ctrl/MEF 96 Treated

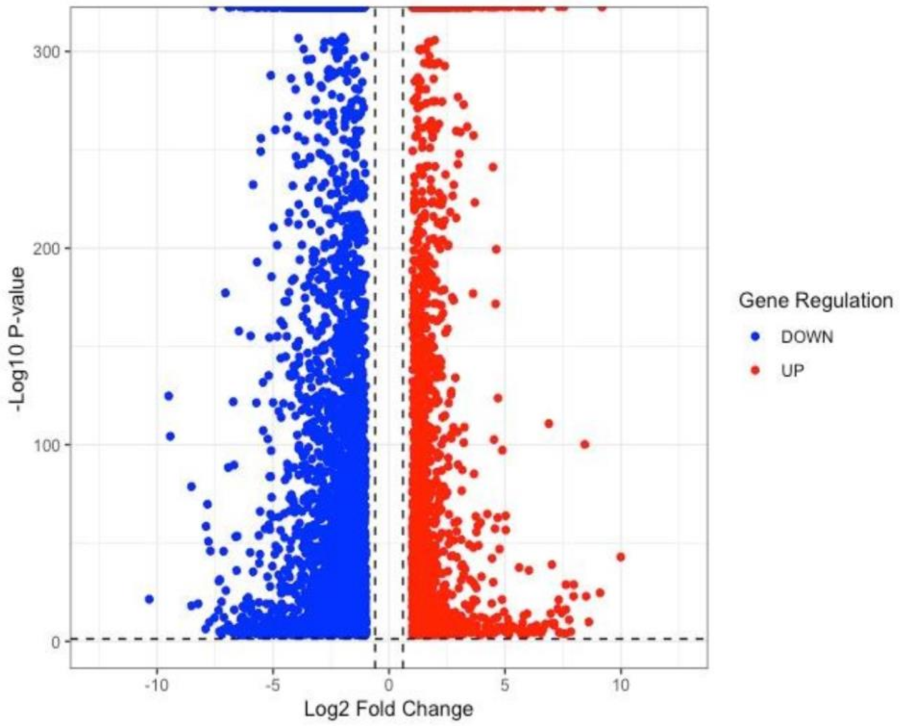

3F: Heatmap of DEPS nPR response to PRG

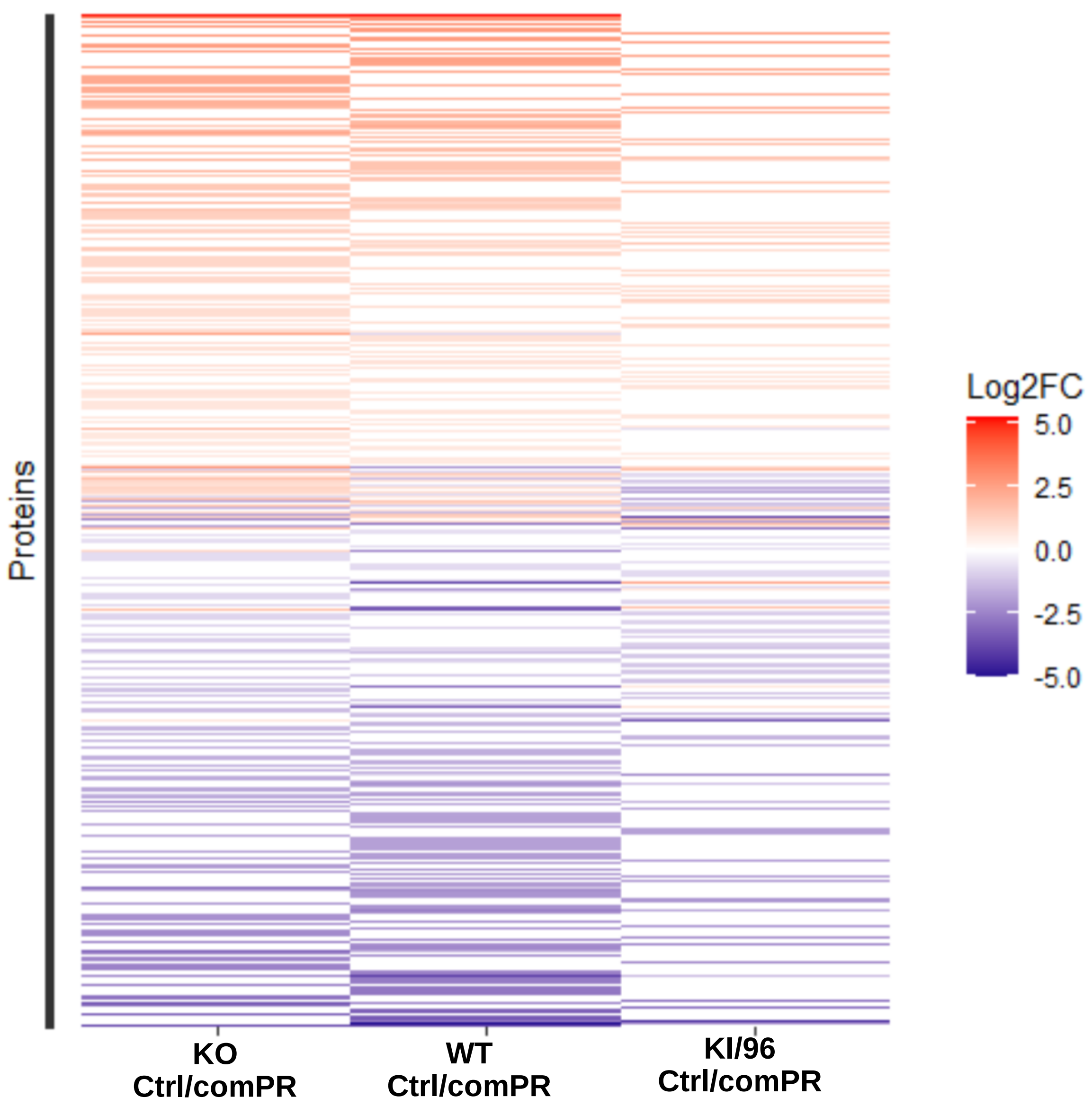

3G. Heatmap of differentially expressed RNAseq nPR response to PRG

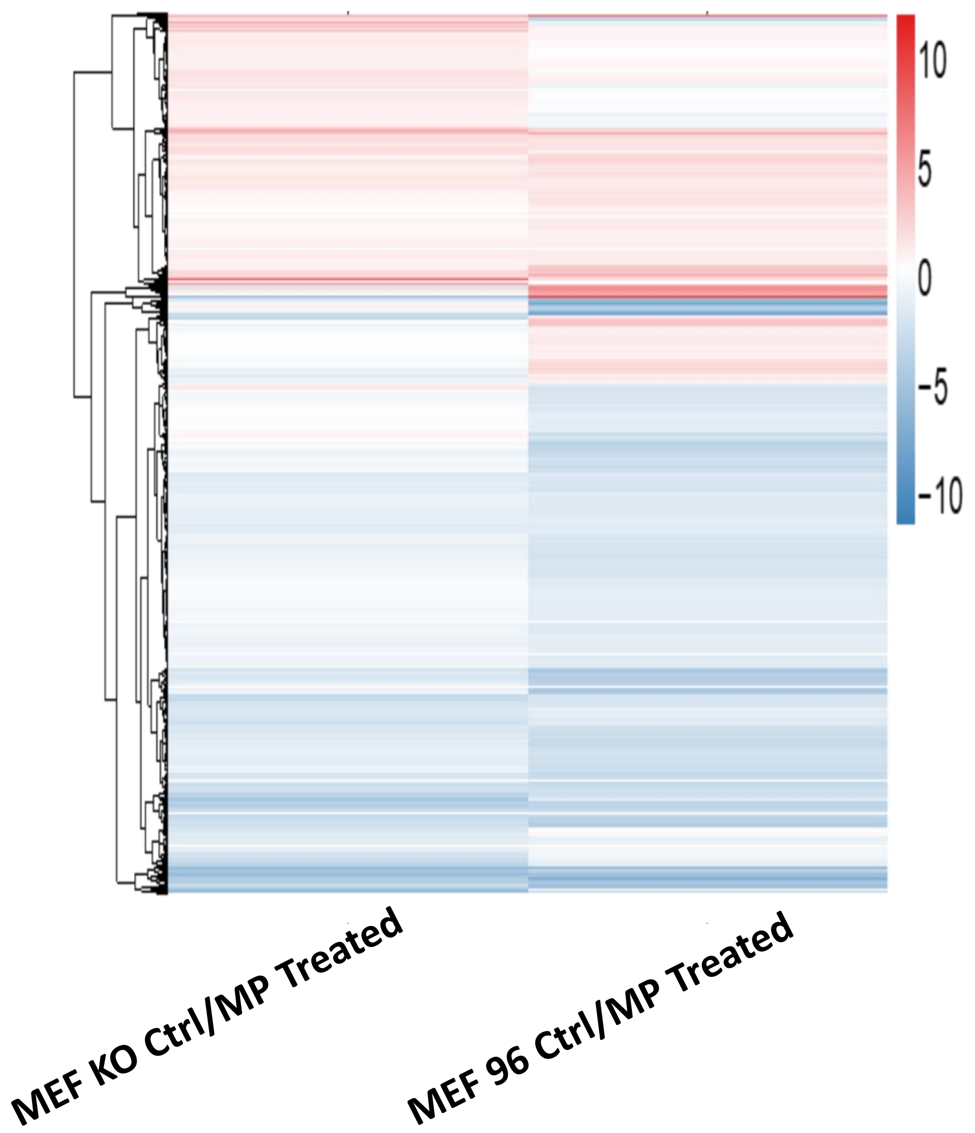

Figure 3H: Proteomic GO nPR

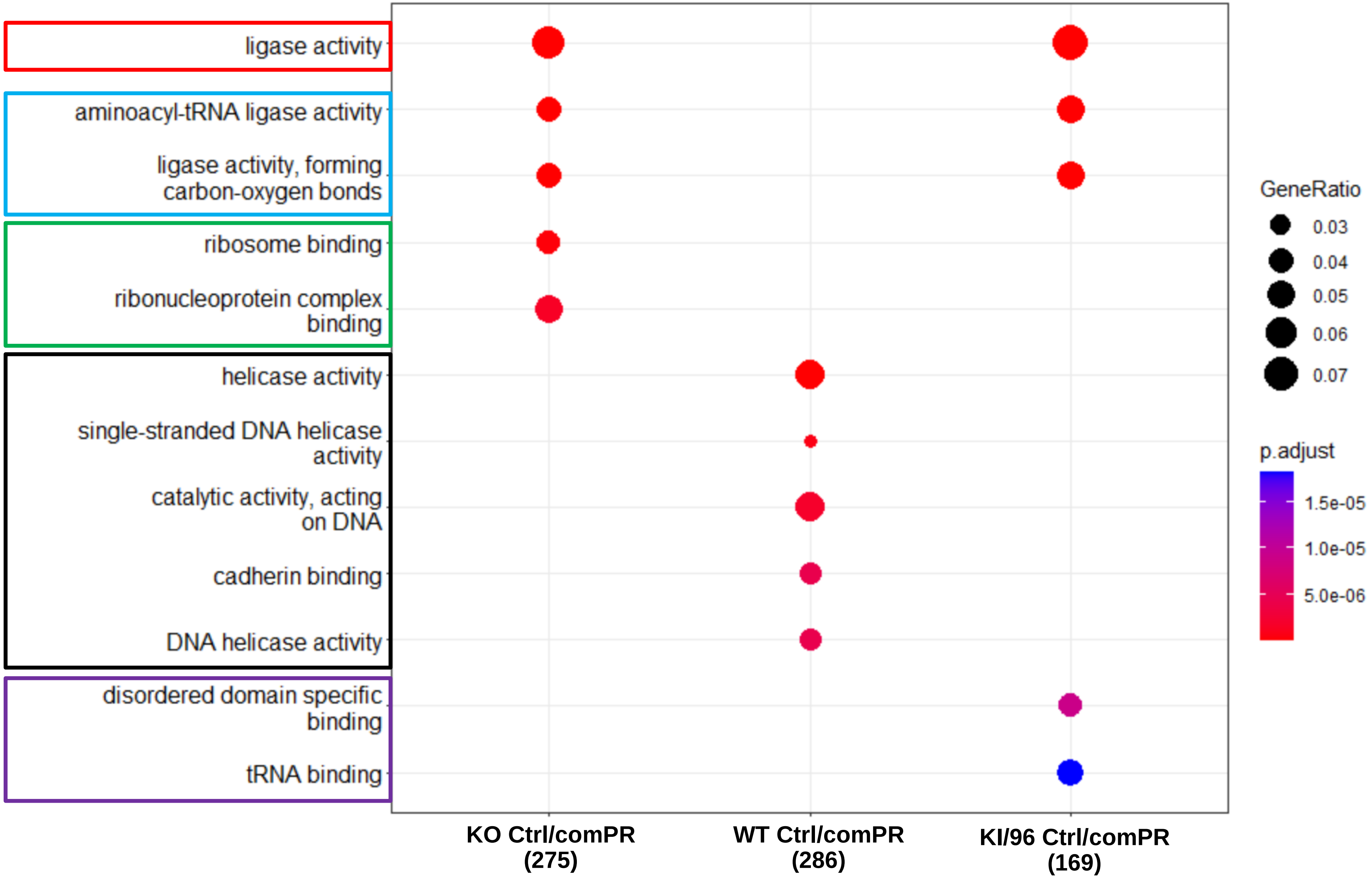

Figure 3I: RNASeq GO nPR

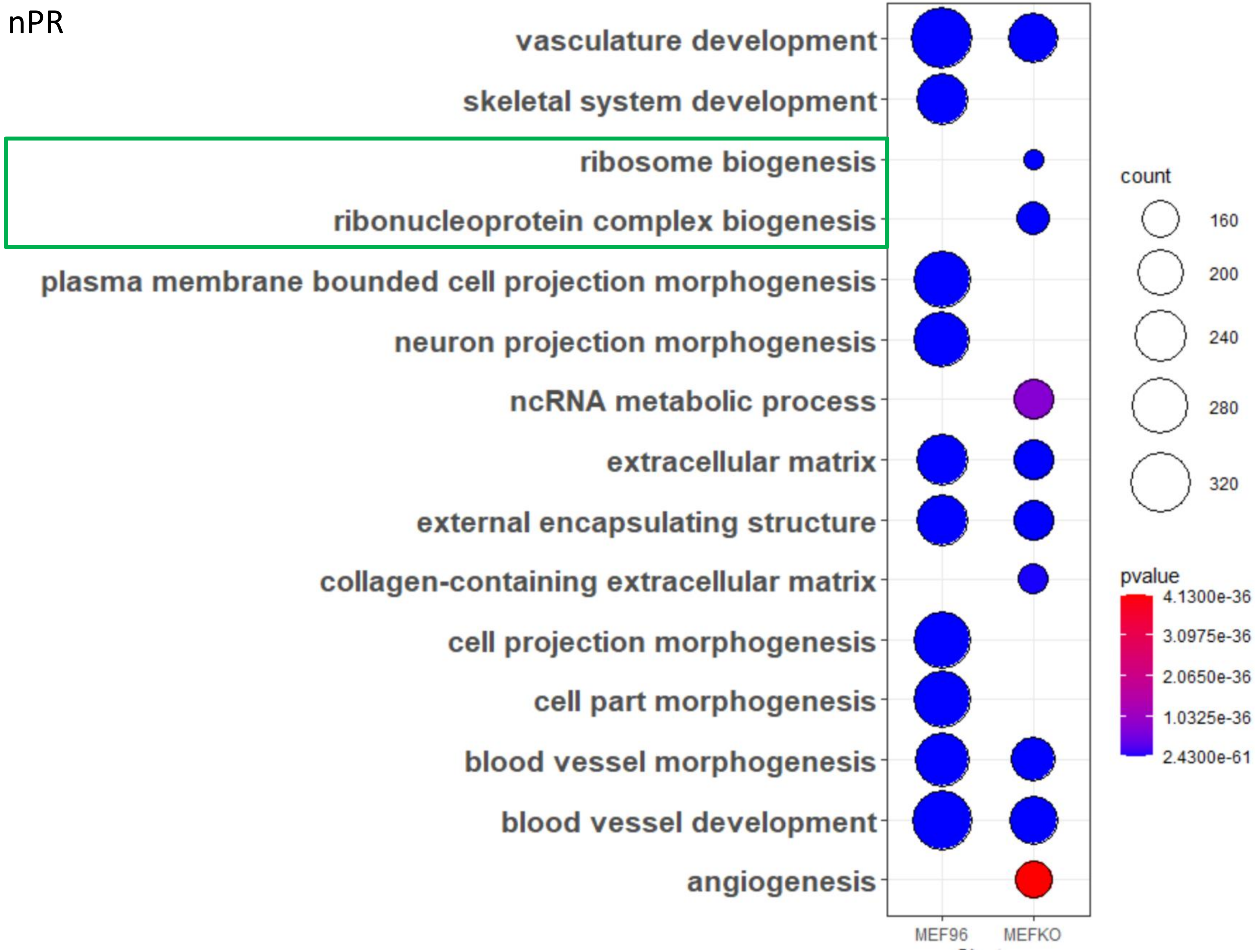

Suppl. Figure 3J: Proteomic KEGG nPR-modulated PRG actions

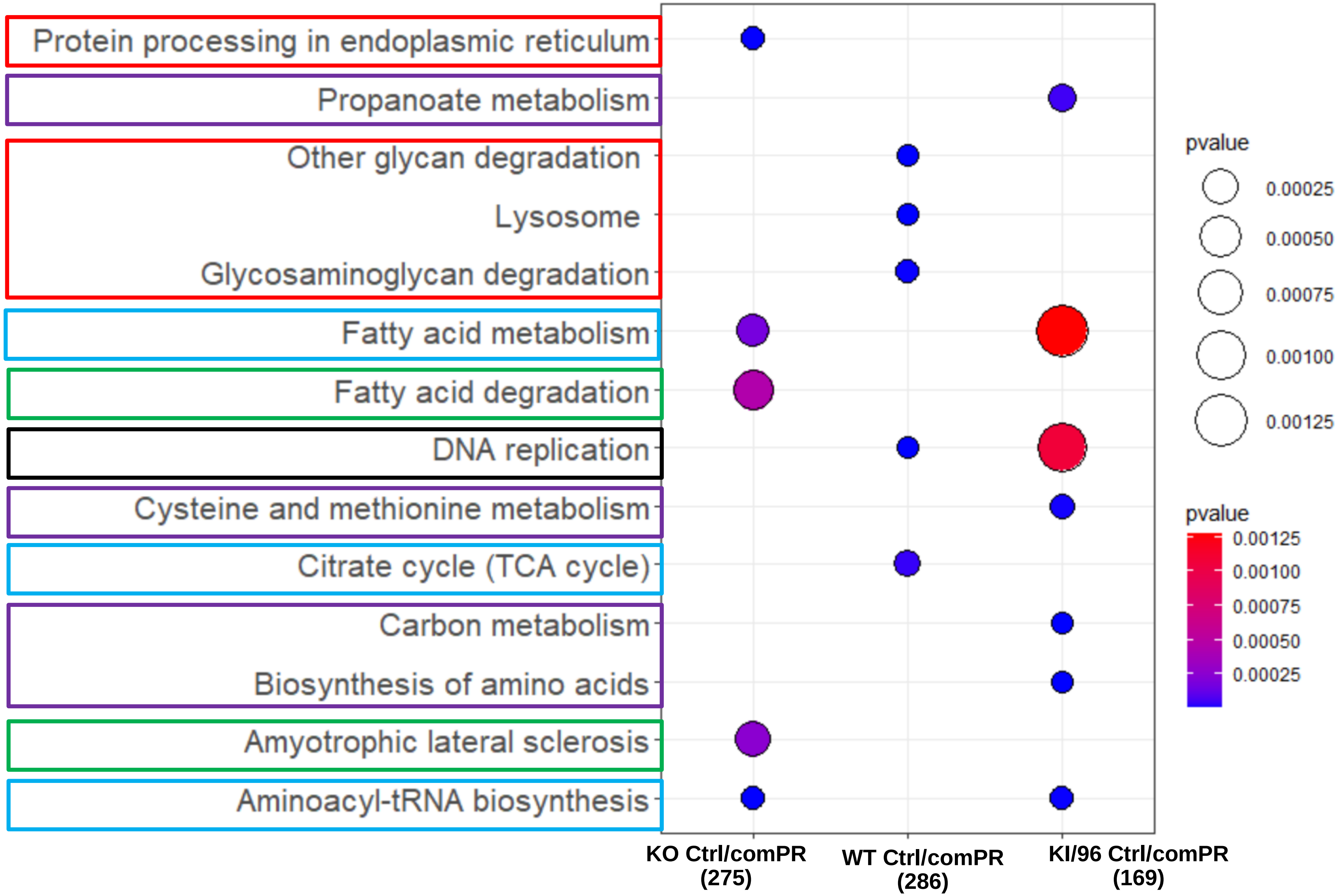

Suppl. Figure 3K: RNAseq
KEGG nPR-modulated PRG actions

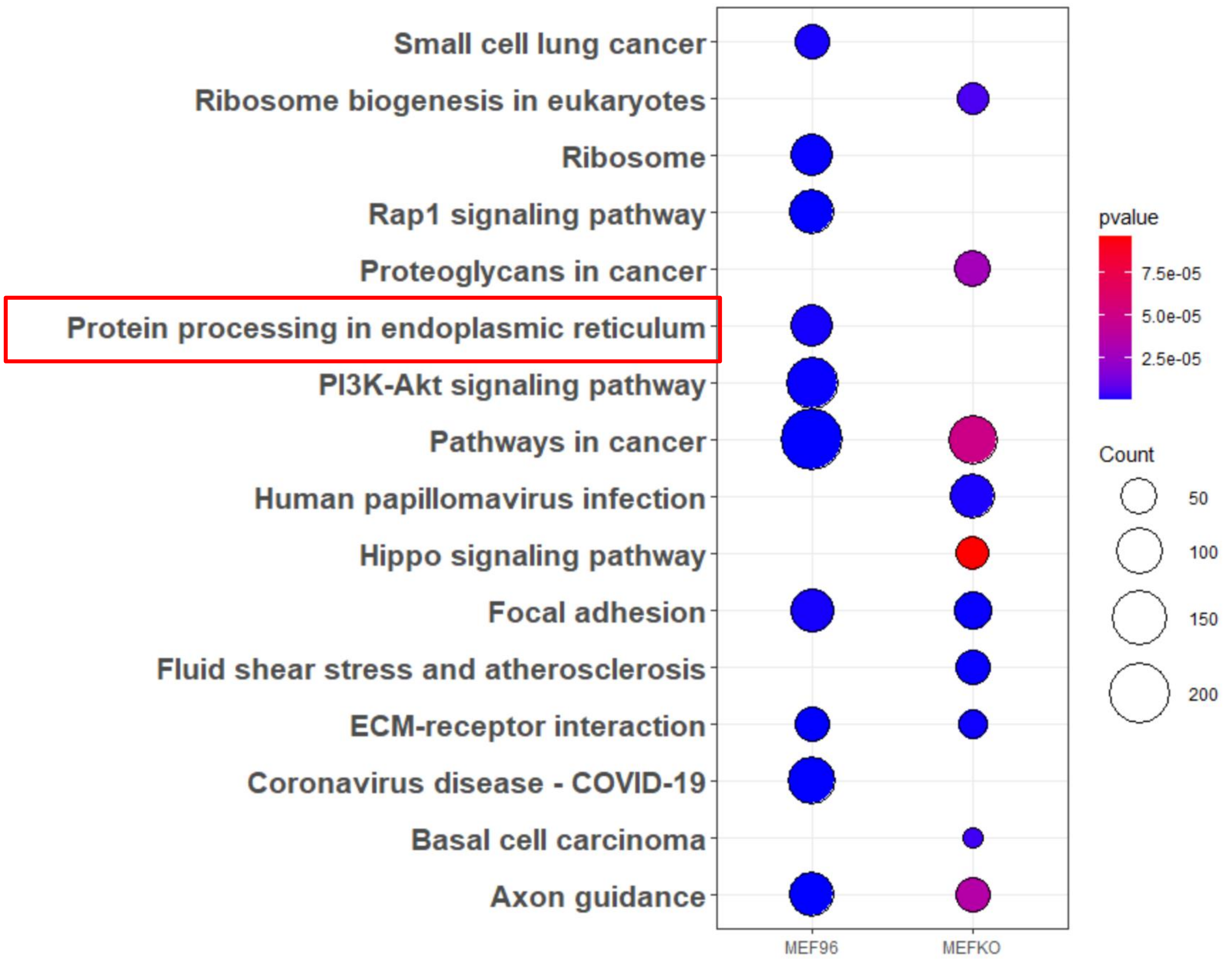

Suppl. Fig. 4. Differentially Expressed Proteins and RNAseq mPR specific response to PRG

Figure 4A: Differentially Expressed Proteins: Membrane Receptors

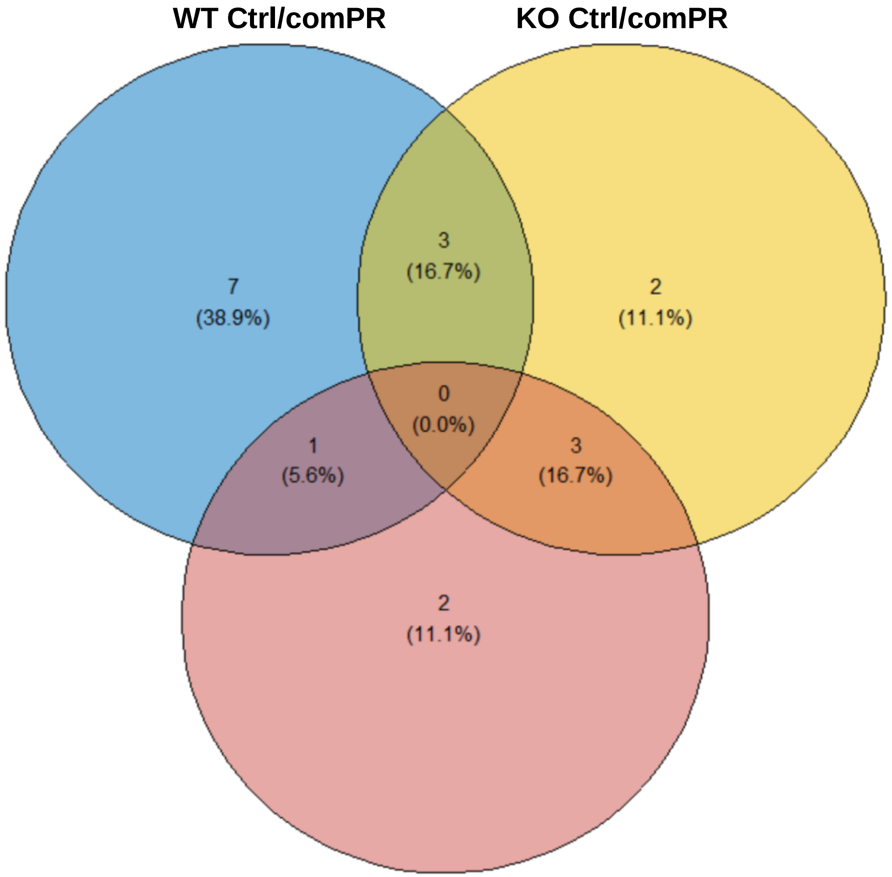

Figure 4B: Differentially Expressed RNAseq: Membrane Receptors

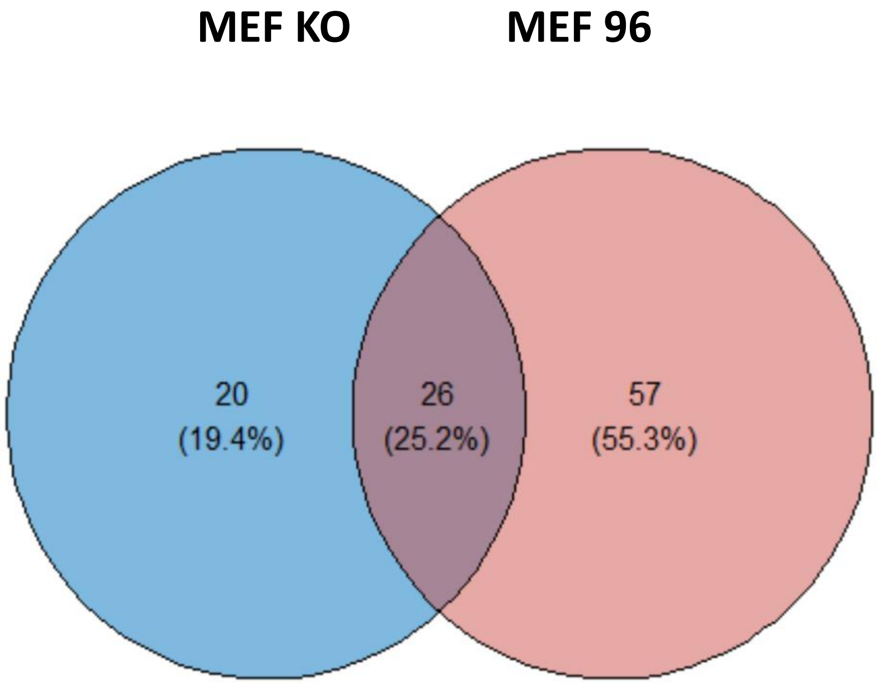

Suppl. Figure 4C: DEP Heatmap mPR response to PRG

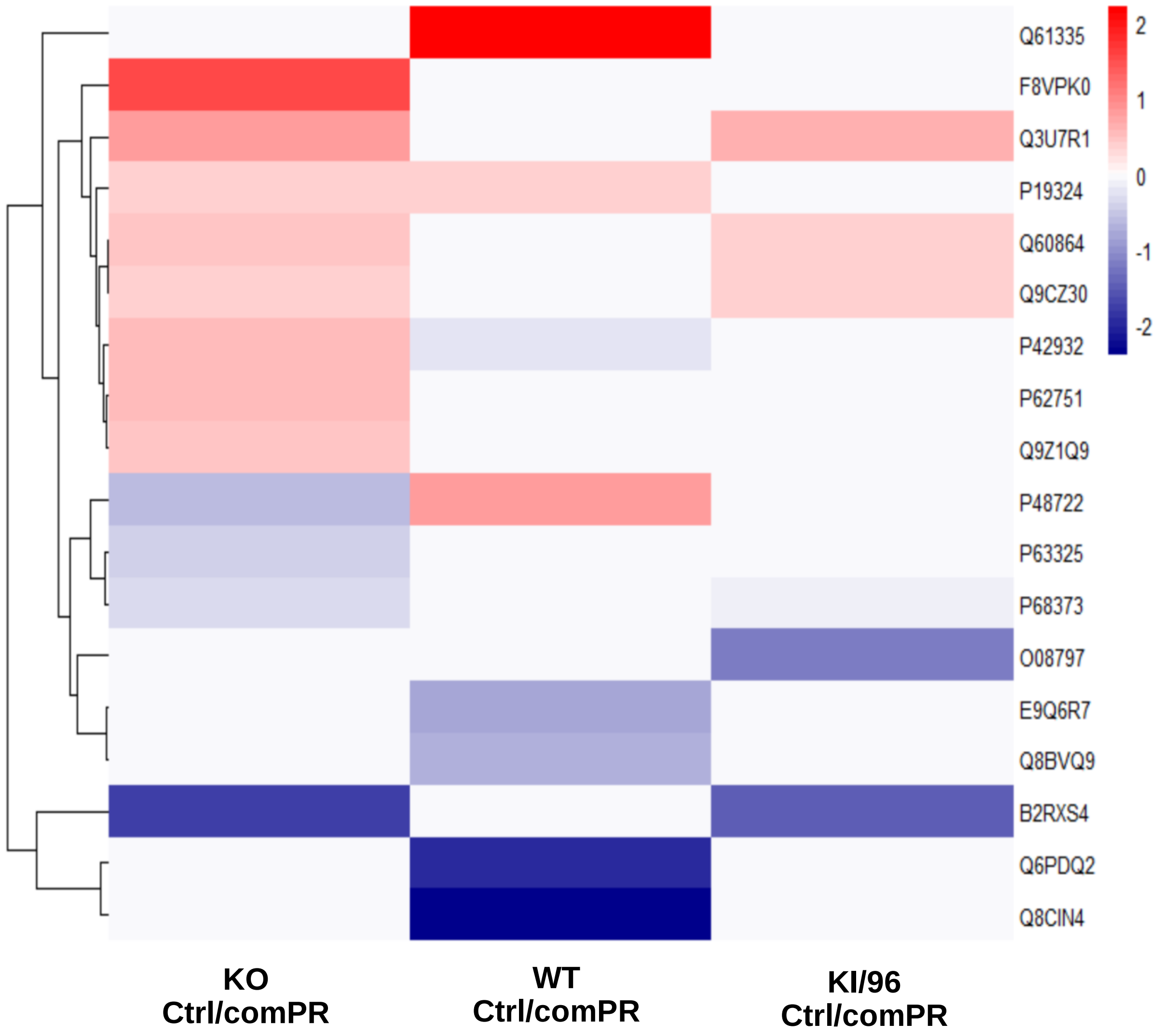

4D: RNA Heatmap mPR response to PRG

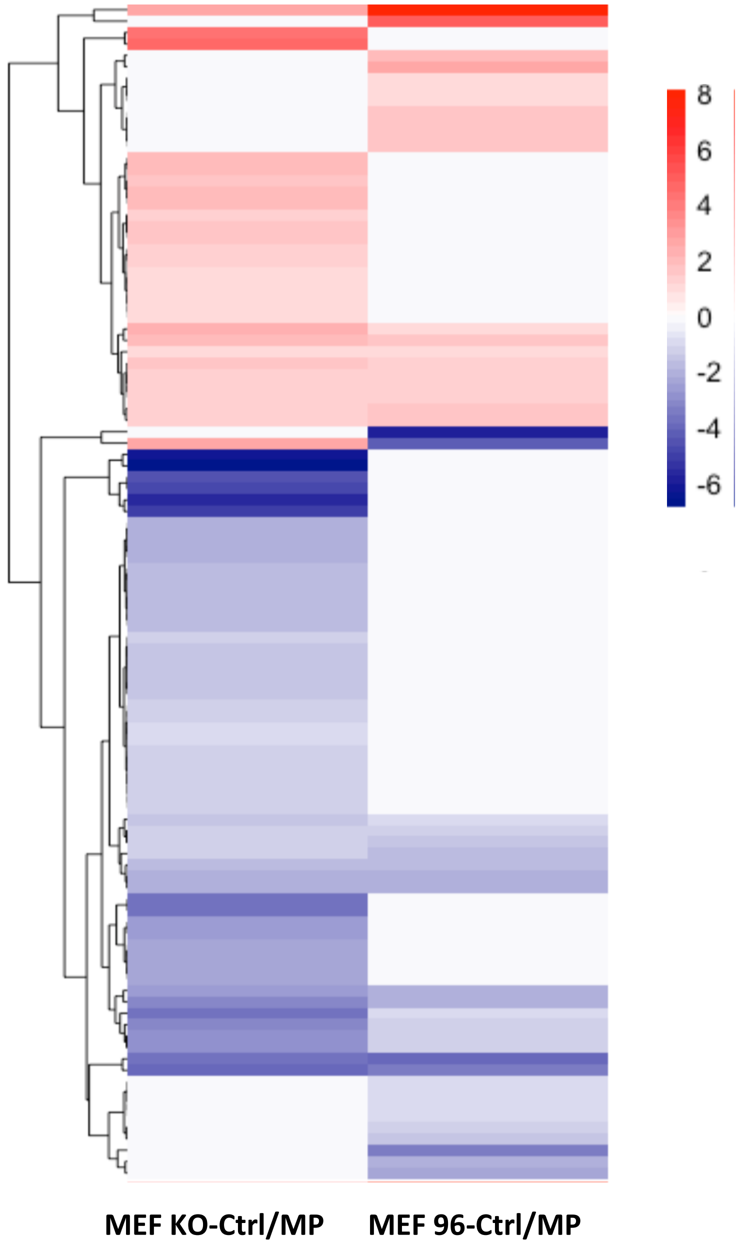

Suppl. Figure 4E: Combined PRG specific action modulated through CCM1 Proteomic enrichGO

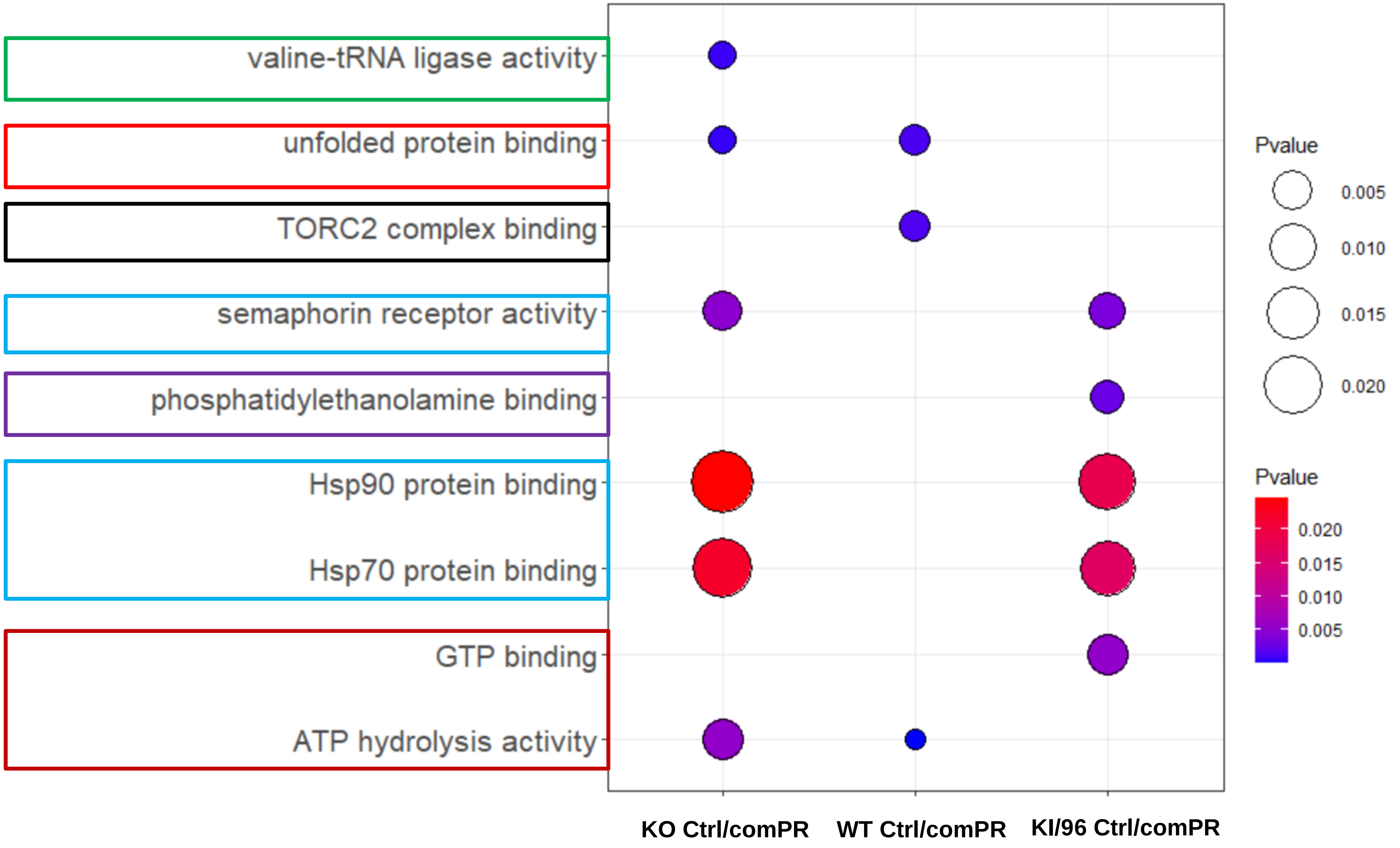

Suppl. Figure 4F: mPR specific PRG RNAseq GO

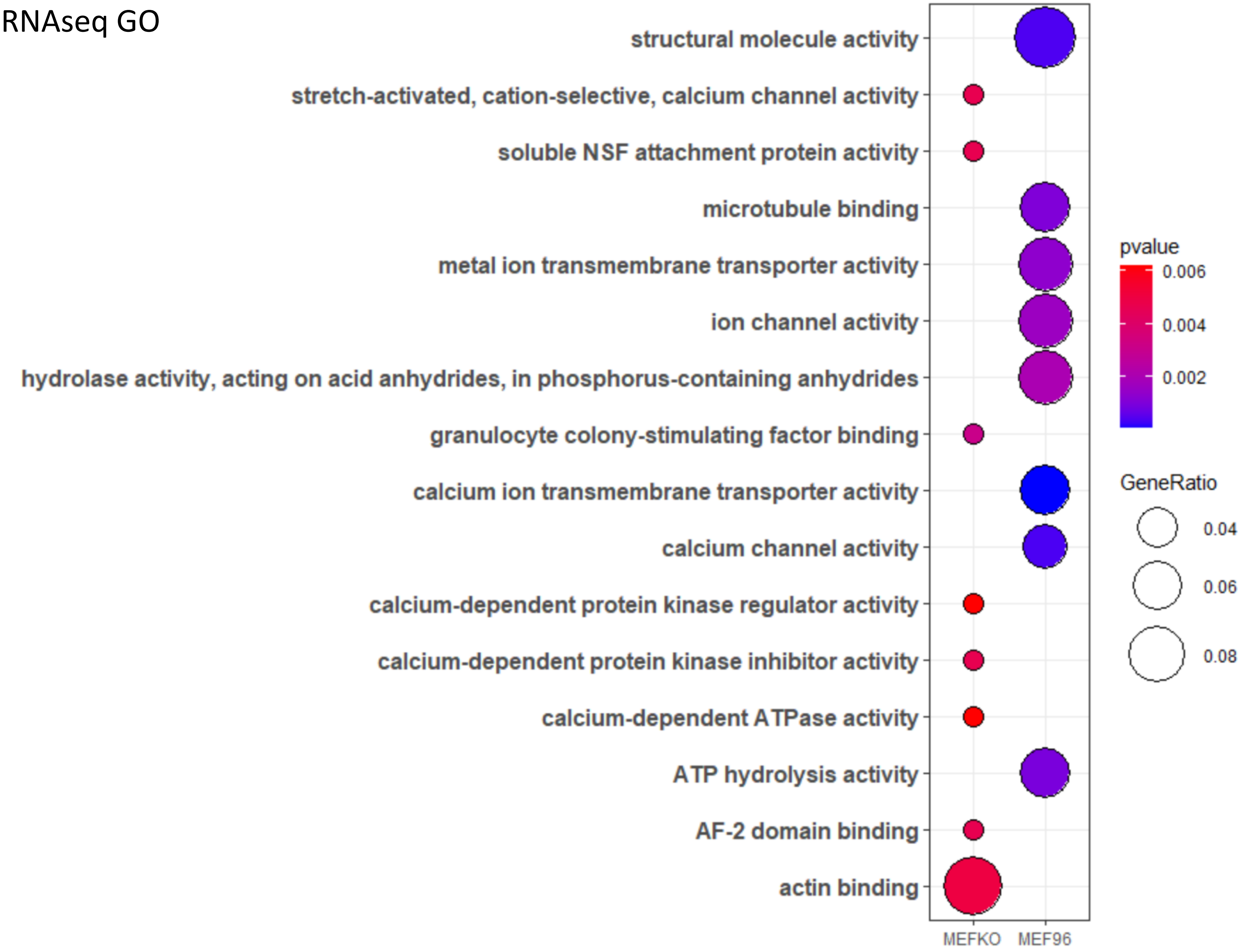

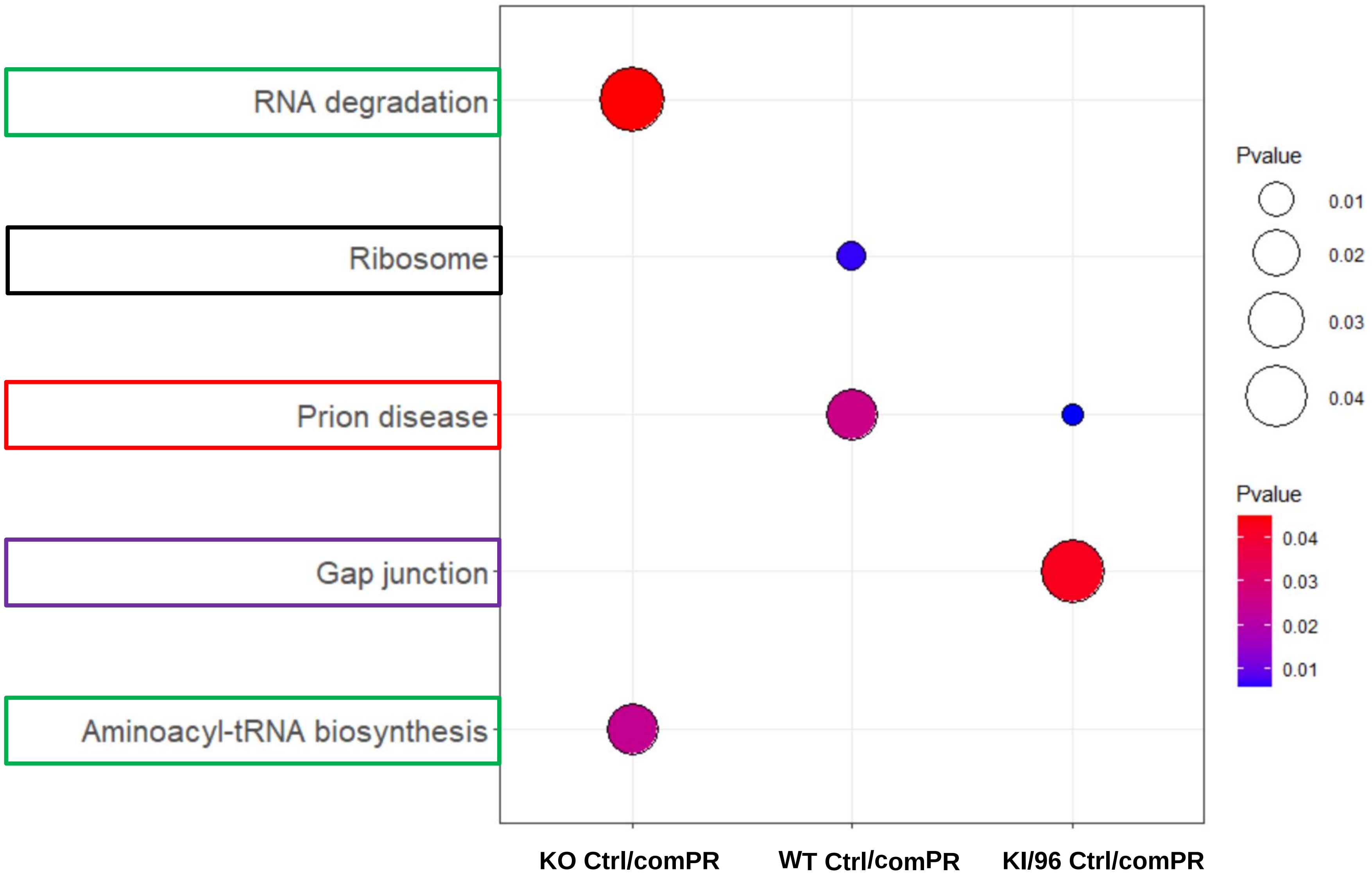

Suppl. Figure 4G. combPRG specific action modulated through CCM1 Proteins enrichKEGG

Suppl. Figure 4H: mPR specific PRG RNAseq GO

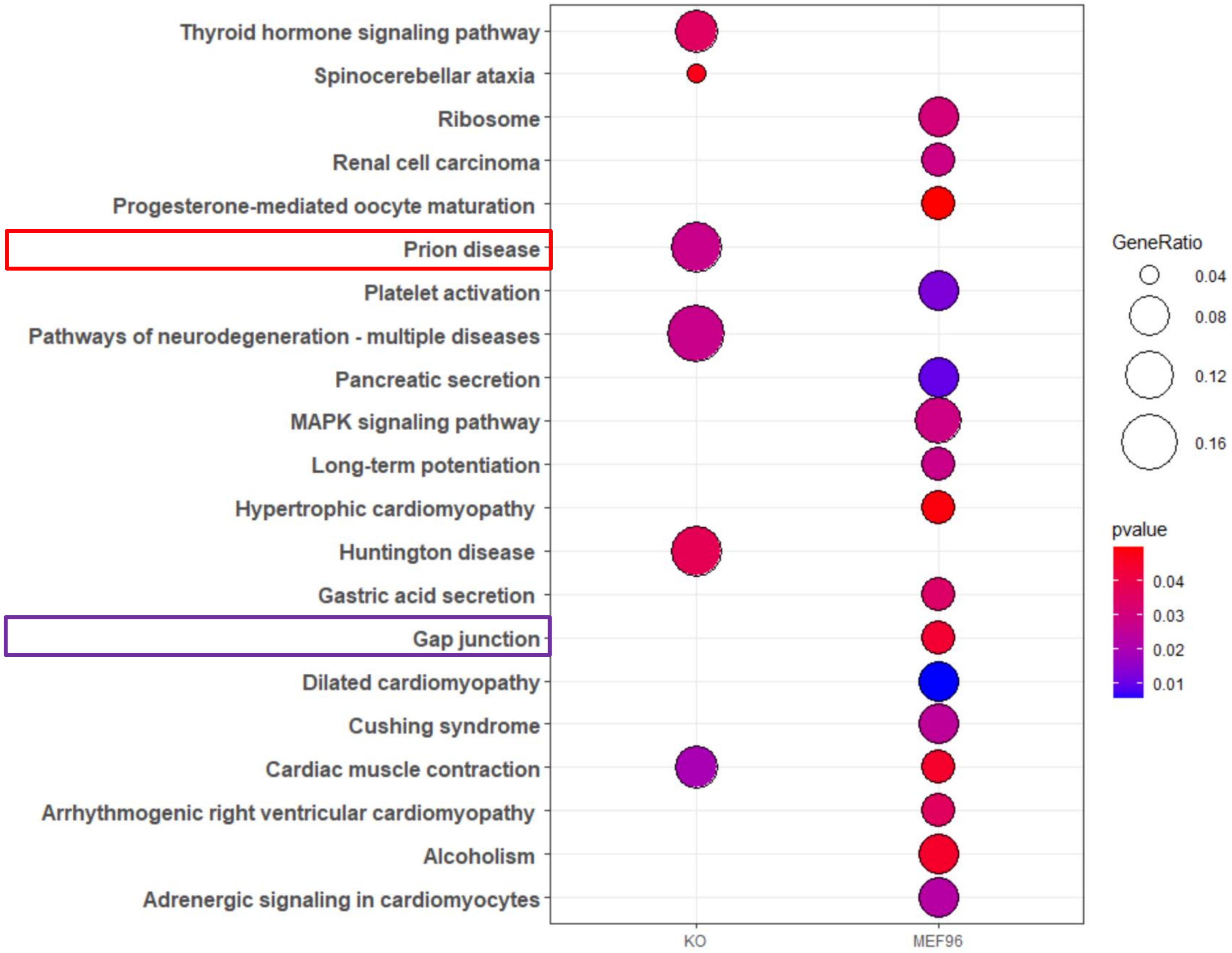

Supplement: Supplementary file 1 [file diagnostics-14-01895-s001.zip › diagnostics-3128947-supplementary.pdf]
